# Food and exercise practices among British Pakistanis; how can Bourdieu’s theory of practice help to understand them?

**DOI:** 10.1177/17579139241270754

**Published:** 2024-10-25

**Authors:** B Hussain, I Shaw, S Timmons

**Affiliations:** Nottingham University Business School, University of Nottingham, Jubilee Campus, Nottingham NG8 1BB, UK; School of Sociology and Social Policy, University of Nottingham, UK; Nottingham University Business School, University of Nottingham, UK

**Keywords:** Bourdieu’s practice theory, food practices, exercise practices, cardiovascular diseases (CVDs), Pakistani, UK

## Abstract

**Objectives:**

The prevalence of cardiovascular diseases (CVDs) are significant among the Pakistani ethnic group in the UK. Existing literature has identified food and exercise practices as contributing factors. This qualitative inquiry investigates food and exercise practices among this group. The study also identifies any cultural resistance to changing prevailing unhealthy practices.

**Methods:**

Five qualitative semi-structured interviews with local Pakistani community leaders, two focus group discussions, and 40 individual interviews with participants of both genders. Bourdieu’s theory of practice was used to analyse the data.

**Results:**

The lifestyle choices of the participants mainly follow the cultural practices of their home country. In particular, three cultural phenomena might have been contributing to CVDs among this community: a culture of consuming fatty and calorie-dense food, complexity in joint decision-making among family members, and a lack of motivation and cultural support for healthy physical activities, especially among women and older adults.

**Conclusion:**

It would be challenging to significantly influence this unhealthy lifestyle in the short term. Integrating religious discourse within health promotion, adopting a whole-family approach, and working with the community on healthier cooking and making exercise options culturally relevant could be helpful for reducing the prevalence of CVDs among the Pakistani community in the UK.

## Introduction

It is estimated that 2.7% (1.6 million) of the UK population associate themselves with the Pakistani ethnic group.^1^ Compared to white ethnicity, South Asians in the UK are at higher risk of myocardial infarction (67%) and ischaemic stroke (29%).^2^ This group also has a higher prevalence of diabetes and hypertension;^3^ risk factors for cardiovascular diseases (CVDs). A systematic literature review and meta-analysis of observational studies on cardiovascular disease-related mortality inequalities between South Asian and White ethnicities in developed countries^4^ found that South Asians had a significantly increased risk of cardiovascular disease mortality compared to Whites (risk ratio = 1.32; 95% credible interval = 1.14 to 1.54). According to the British Heart Foundation (2010), about 25% of deaths of first-generation Pakistanis in England and Wales were due to heart diseases. This is a significant issue to address from a public health perspective.^5^ It appears that the higher prevalence of CVDs among Pakistanis is sustained. As Netto et al.^6^ noted 15 years ago, CVDs were higher among Pakistanis in the UK in terms of mortality and prevalence.

A qualitative synthesis of studies on cardiovascular disease prevention among Pakistani communities in the UK identified that social and cultural practices (such as family dynamics, and a lack of cultural and social support for preventive measures such as healthy diet and exercise) among this community affect their cardiovascular disease prevention behaviour.^7^ In the UK, a study conducted among South Asians (including Pakistanis) found that the participants attributed the cause of their CVDs to their food practices.^8^ However, there was also a significant number of participants who also attributed the cause of CVDs via their religious beliefs. Attributing CVDs to religious beliefs meant these participants believed that they had CVDs because Allah (God) wanted them to experience this condition and not from their own actions. Similar results were found by Netto et al.^6^ They argued that in order to make persistent lifestyle changes, the cultural, social, historical, environmental, and psychological forces that influence health behaviour first need to be addressed. Despite the size of the British Pakistani community, and substantial health issues, they remain under-studied. An exception to this is Kokab et al.^9^ who studied British Pakistani men and highlighted the importance of social networks and capital, particularly Pakistani-run gyms, and the mosque. Bush et al.^10^ found that traditional family hospitality also plays an important role in the life of South Asian people. Traditional South Asian hospitality involves offering a lot of food to guests, especially meaty, rich, and savoury dishes. This tradition of hospitality then contributes to the consumption of unhealthy food among the Pakistani community.

The review by de Morais Sato et al.^11^ identified that Bourdieu is helpful in understanding eating practices among different social groups, though the focus of the articles they reviewed was principally on social class, suggesting that ethnicity remains under-studied from this perspective. Similarly, Blue et al.^12^ and Noor et al.^13^ noted that Bourdieu offers a useful approach to study the relationship between micro- and macro-level antecedents in relation to health. These theories offer a relational approach through which researchers can examine the material and discursive entanglements that inform the forms, meanings, and experiences of social practices such as food and exercise practices and their health impacts.^12^ Using Bourdieu’s concept of cultural capital, Oncini and Guetto^14^ found that social factors such as familial cultural resources play important role in shaping children’s food choices.

## Bourdieu’s Theory of Practice

Among the reasons why diet and exercise practices have proven so resistant to public health intervention is their complexity and their deep interrelations with the wider social context. Individuals are not the autonomous agents that many believe them to be; they are constituted and influenced by the multiple social contexts (such as family, home, work, school, community) they inhabit. Social science has struggled to provide broad and persuasive explanations of social action (and inaction). An exception to this is the subtle and nuanced approach of French sociologist Pierre Bourdieu. Rather than attributing social action either to individual choices, or to the influence of social context, Bourdieu finds a way to keep both in play. For Bourdieu, the relationship between his concepts of habitus, field, and capitals (all explained below) solves the problem of understanding social action, and thus determines the nature of practices. These concepts are to be used as a ‘flexible and open analytic framework’^15^ to guide social scientific research rather than sort findings into analytical categories. For Bourdieu,^16^ practices are a product of the relationship between habitus, field, and capitals.

Habitus refers to the internalized dispositions, habits, and ways of thinking that individuals acquire through socialization within their specific social context. The habitus shapes individuals’ tastes, preferences, and behaviours, including their food choices and exercise patterns. Individuals from different social classes have different habitus, which are shaped by their class position and the cultural resources available to them. These differences in habitus can lead to variations in food behaviours and exercise pattern between different socioeconomic groups.^17,18^ Bourdieu also emphasizes the role of social capital in shaping practices such as food and exercise. Social capital refers to the resources, networks, and relationships that individuals possess, which can provide them with access to certain opportunities and benefits. In the context of food and exercise behaviours, individuals with higher social capital may have greater access to information, resources, and social networks that enable them to make healthier food choices and engage in exercise practices that promote well-being. It has been demonstrated in several studies that the theories of practice are not only an important tool to understand food consumption intellectually but to achieve food-related social change as well.^19–21^ In Bourdieu’s approach, a field is a particular social space with its own unique characteristics.

Kandt^22^ uses a Bourdieusian approach to study the relationships between lifestyle and health inequality, again showing that ‘behaviours’ are not individual choices, but social phenomena. Food practices are thus as much, if not more, a social phenomenon, than a product of individual decisions. One of the virtues of the practice theory approach is that it provides causal linkages between wider social phenomena such as poverty or racism, and their consequences for individuals in terms of ill-health, without reverting to psychological explanations of individual behaviour, which have been shown to be limited.

Warde^23^ distinguishes between versions of practice theory, which ‘claim that doing precedes and steers thinking that habit and routine are the fundamental basis of all action’ and weaker versions where ‘explanations should give due credit to routine, know-how, shared understanding, the embodied and the material’ in accounts of the social world. We align ourselves with the weaker formulation in this article. Warde^23^ further calls for how practice theory ‘might be employed to address problems of description, interpretation and explanation of social processes and behaviour in a particular domain’, rather than further theoretical elaboration. Bourdieu suggested that practices such as food and exercise are culturally inherited rather than innate.^16^

We agree with Warde^24^ that traditional approaches to health promotion operate with an often-unacknowledged assumption that human beings are fully rational and regularly take conscious decisions about food and eating, minimizing the roles played by habit, tradition, and community. Ironically, this is confirmed by research in psychological disciplines themselves (such as Neal et al.^25^).

Using Bourdieu’s theory of practice as a lens, this study explores food and exercise practices among the Pakistani ethnic group living in a UK city. The study also aims to identify any cultural resistance to changing prevailing unhealthy practices.

## Methods

In line with the recommendation to use qualitative methods for public health research,^25^ we used three qualitative tools to collect the data. The data collection was:

1. Individual semi-structured interviews (*n* = 5) with community leaders. These interviews were helpful in getting background information about the community and accessing participants. The leaders were selected through personal recommendation and snowballing.
2. Separate focus group discussions (*n* = 2) with male and female community members.
3. Individual semi-structured interviews (*n* = 40) with males (*n* = 20) and females (*n* = 20) of Pakistani origin. These participants were selected through snowballing.

The data collection took place in a UK city where there is a substantial British Pakistani population. The characteristics of the sample is given in Appendix 1, which shows that we were able to recruit a diverse sample. All the participants who were approached agreed to participate in the study at a mutually agreed time for the interviews. Focus group discussions were undertaken in local community centres, whereas individual interviews were conducted in homes of the participants. The data were collected by the first author who has the relevant research, linguistic, and cultural skills. The data were digitally recorded and transcribed verbatim by the first author. All data were collected face to face. Individual semi-structured interviews were 40–60 min long, whereas focus group discussions lasted for 60–70 min for both groups. Separate topic guides were used for focus group discussions and semi-structured interviews.

The data were analysed using the cognitive mapping technique.^25^ Data were coded by first and second authors and regular meetings were held with the team to explore the themes emerging from the data, which helped to develop subsequent interviews in a process of analytic induction.^26^ Themes were derived from the data. We did not use any computer-assisted qualitative data analysis software such as NVivo. Participants’ quotations along with their gender and participant number (e.g. M15 means male participant number 15, F2 means female participant number 02) are presented to illustrate the findings. Owing to time limitations, we were unable to share transcripts with the participants. However, the first author was invited by a local ethnic radio station to share study findings in a radio programme.

## Findings and Discussion

### Food practices

#### Defining typical Pakistani food

All participants were of the view that typical Pakistani food is fatty, spicy, and rich in calories. The two main components for a basic Pakistani meal are Roti (Chapati) and Salin (Curry/soup). Occasionally Roti is replaced or joined by a rice dish. Both Roti and/or rice are eaten with some type of Salin, which is made of vegetable, lentils, or meat.

*‘Typically Pakistani Food is “Roti Salin” (Chapati and Curry) . . . It is chicken Salin, gosht (mutton), Daals (beans), fried eggs, and . . . like I mean. Biryani (rice with meat)’.* (M15)

*‘Typical Pakistani food is rich, very tasty, fried most of the time and very full of calories’.* (F2)

All the participants categorized typical Pakistani food as unhealthy. They were of the view that even if the contents of the food are healthy, the way it is cooked makes it unhealthy. The participants also reported that in the Pakistani community food is often over-cooked, which means if 30 min of cooking is required for a vegetable to make it edible, people in the Pakistani community may cook that vegetable for 45 min.

*‘I think certain parts, it is healthy. I mean meat. It is the way it is cooked I think is the problem. [. . .]. That is the amount of salt and spices put in or the oil used. I think that is quite excessive and makes it unhealthy or letting meat cooked such a long time that a lot of sorts of nutrients in the meat are sort of evaporated. I think in terms of the contents put in, the vegetables, tomatoes, it is healthy food*’. (M15)

Use of excessive oil in cooking is linked to the perception that it makes food presentable and acceptable to family members. The participants were of the view that it is difficult for them to change cooking practices, because if food is not cooked in the expected way, then it will not be acceptable to family or community members.

#### High consumption of meat and sweets

The other things that participants thought made typical Pakistani food unhealthy was consumption of meat, fried food like samosas and pakoras, as well as traditional Asian sweets (Meethai). With a few exceptions, all the participants were of the opinion that level of meat consumption was very high among the Pakistani community.

*‘It [Meat consumption] is very high in the Pakistani community’*. (M15)

Meat dishes were considered as high status. Offering only vegetable or lentil dishes to the visitors or at social events was identified as something that would not be acceptable. Guests would feel insulted and not well looked after.

*‘It [meat] is a premium dish compared to daal (lentil). You know if someone comes, you do not make daal, you make gosht (meat). And some people I think now because they can afford it, they want [meat] every day’.* (M15)

Meat consumption is a masculine phenomenon in this community. The female participants were of the view that male family members tended to like more meat than females. They linked this with masculine strength. There was, however, some indication that meat consumption was decreasing in second and third generations.

*‘Pakistani and also Muslim, is meat eater . . . We eat lot of meat. Male eat lot more [because] it gives strength to his muscles and bones’*. (F15)

*‘But there is a difference in men among second generation. First generation was more meat oriented’.* (F3)

There was also a perception that meat is more affordable and easier to cook than vegetables. As many of the participants originally came from poorer backgrounds in Pakistan where meat is expensive, this might be a reason that when meat has become affordable, they eat it more. ‘*When it is available and affordable, its consumption has increased in this community’* (F5).

#### Food and gender

Food preparation and cooking at home is traditionally a woman’s task. Menus were decided in consultation with other family members, but the final decision was made by a woman (wife or mother). This consultation was an effort by women to increase the acceptability of what was cooked among all family members. This decision was normally made at breakfast.

#### Food and ethnic identity

The majority of the participants associated their ethnic identity with typical Pakistani food. The association of food with ethnic identity was complex, including the way food was cooked and presented, the type of dishes cooked, and the origin of these dishes. These were specific to specific ethnic groups and their place of origin.

*‘Yes, it is obvious, for example me, who is here for 40 years, instead of this long time, I will continue my food, whatever, it is curry and chapati. As a Pakistani, I would like traditional food. Although, I am suffering from heart problem, but still, I would eat my food, it may be bit light food, cooked in my own way. Asian food is our identity*’. (M9)

*‘If you make some English food, people [guests] will talk . . . they will say you are losing identity’.* (F5)

There were, however, four participants that did not see any link between the food choices they made and their identity.

*‘I never thought of eating Pakistani food because of Pakistani. I have just always grown up with eating it and see it as normal . . . I do not associate my identity with the food’.* (M15)

#### Food choices on social and religious events

There was a very clear view that social and religious events such as weddings, *Khatam* (at a death), and Ramadan influence food choices. Typically, these events have set menus, but choices were also related to income. There would be a traditional menu but if someone had money and wanted to spend it, he or she will add other dishes. In Ramadan, particularly, consumption of fried food increased.

*‘I think Ramadan is worst month. Samosa, pakoras, there is perception that we need to have it on the daily basis’.* (F3)

*‘In Ramadan . . . in my house . . . When you come to iftar . . . there is so many dishes on the table, so many dishes like, if you eat them, it is totally full for Taraveeh (night prayer during Ramadan)’.* (M15)

Public health research has consistently identified the directly proportional relationship between socioeconomic disadvantage and poor health. However, the public health field has generally looked at food and exercise behaviours from the perspective of epidemiological or population health, mainly relying on quantitative methods. Similarly to Kokab et al.,^9^ we also found that there is a complex hierarchy of socio-cultural and religious factors that influence food choices by members of the Pakistani ethnic group, where health may not be a priority.

Our study also found that energy dense food is eaten by members of the Pakistani community. This is similar to evidence found by Gupta et al.^27^ in the US context who noted that among South Asians (including Pakistanis) the main dietary trend after migration is a substantial increase in energy and fat intake, and a switch from whole grains and pulses to more refined sources of carbohydrates, resulting in a low intake of fibre and vegetables leading to obesity and cardiovascular health risks.

More widely, our research shows that food practices arise from and in a complex world (for Bourdieu, field, capital, and habitus). Tradition, ideas about family and hospitality, ethnic identity, and gender (all what Bourdieu would term habitus) intersect to give rise to the practices we have seen and analysed. No one factor explains them; and thus there is no one intervention that could begin to alter them.

## Exercise

The study found the exercise in this community is also embedded in the originating cultural context. The majority of participants reported that they do not do any kind of regular physical exercise, and this was especially the case for women. There were many reasons stated for this including understanding that they needed it, lack of time, cost, and age. However, a commonly given reason was a lack of motivation and a ‘culture of laziness in the community’ (M13). The participants considered slow walking, offering prayer five times daily, and doing household chores as enough exercise.

*‘I do not do any exercise . . . I have not got those calories to burn. I got kids; looking after them is good exercise’.* (F17)

*‘It is laziness, people do not exercise. Motivation is lacking . . . if you are overweight, they will not feel bad . . . even wife do not say anything . . . Women who come from Pakistan are worse in terms of exercise . . . after marriage women do not take exercise seriously’*. (M13)

The study found that there were many cultural barriers for both men and women for exercise, particularly use of gyms or leisure centres. These cultural barriers were more pervasive for women. The cultural barriers included not being willing to join mixed gender gyms, wearing exercise clothes, and the pressures from caring roles within household.

*‘In Pakistan women do not exercise culturally . . . they are used to that . . . when they come here . . . They do not’*. (M7)

*‘There are not many choices available in the local areas. The local gym has one or two sessions available for women only, when sessions are available, they might be busy with the children’*. (F8)

*‘Swimming centres have women-only sessions, but they have male guard, we complain many times, but they have not changed it. There is no point of having women swimming centre and having male guard on the pool side. I do not go’*. (F8)

The participants unanimously agreed that exercising for women is not inherently anti-Islamic as Islam encourages keeping fit and healthy. They said that women could exercise within the limits of religious teaching, which requires them to be segregated from men and wearing proper dress.

*‘Exercise for women is not anti-Islamic. If it is in a safe environment’.* (F12)

Here we can again see an analogous picture of the complex intersection of social and cultural capital combined with habitus produces the pattern of practice (or rather the lack of it) around exercise for health. It is possible that there is also a historical component to this. The British Pakistani community tends to have migrated from poor rural areas where daily life was dominated by hard physical labour (agricultural and domestic).

## Promotion of Healthy Food and Exercise Practices

The Bourdieusian approach relies on understanding that people are not fully autonomous agents, nor are they the unwitting dupes of structure and culture. In order to change practices, it is therefore necessary to engage with habitus, and promote social capital, within the context of the field the people inhabit. We can see what this more Bourdieusian approach to promoting a healthier lifestyle might look like if we consider what our participants had to say about what interventions they thought might be effective. In fact, few participants felt pessimistic about changing unhealthy food and exercise practices among the community, and majority of them gave suggestions.

The need to increase awareness among the Pakistani community about the role of diet and exercise was identified a first step towards bringing change. However, it was important that the presenter should be professional, and speak the various regional languages that are spoken in this community. The participants also suggested incentivising people to achieve good attendance in educational and awareness-raising sessions.

*Team of bilingual people. GP has record of everybody. Maybe they can send worker to visit the families of these people and tell them about healthy lifestyle.* (F8)

*Do you want me to be honest, if you want people to come for cooking session, give them 10 pounds every time they turned up, they will be happy. That is truth.* (F12)

Participants suggested that consistent efforts would be required, and people would not change by just giving them leaflets and a few face-to-face sessions. A team of dedicated people with relevant professional qualifications and experience would be useful.

*Leaflet, home support, visiting people and revisiting them to see whether they have made the changes. There is no point going once and handing them leaflet and coming away because those leaflets will go to dustbin.* (F1)

The role of authority figures such as Imams and general physicians (GPs) was thought to be central in behaviour change towards healthy food and exercise. It was suggested that people could be educated effectively about health issues by linking these issues with religious teaching. Members of the community attend the mosque five times a day, but there is a larger congregation for Friday prayer. Men from almost every household and some women attend Friday prayer. This was seen as an opportunity to make people aware. It was suggested that the Imam could relate healthy eating and keeping fit to religious teaching.

*Imams need to play role incorporating in their Friday sermon. Islam and Sunnah (Prophet Peace Be Upon Him way of doing things) is whole way of life. Prophet Muhammad (PBUH) was perfect example.* (F11)

*Religion needs to be brought in not only on Namaz (prayer) and Hijab but food as well.* (F1)

The research found that role of people who have professional authority such as doctors and pharmacists may be useful. In the COVID pandemic, it was noted that advice from professionals of the same ethnicity was effective in overcoming vaccine hesitancy. It also tends to challenge the fatalistic view that illness is a visitation from God to challenge one’s faith.

*You need doctor to get Pakistani people scare of these things. If doctor say that will work.* (F17)

Current interventions to promote healthy food and exercise practices among the British Pakistani community may not be helpful as these are based on generic and simplistic understandings of existing lifestyle practices among this community.^28^ Food and exercise practices in the British Pakistani community are very complex and deeply interlinked with their culture of origin and migration. In order to promote healthy food and exercise practices among Pakistanis, addressing the sociocultural, religious, and behavioural factors affecting these practices becomes important.^29^

## Conclusion

Constructively, Bourdieu’s practice theory provides a more nuanced understanding of how social inequalities, cultural practices, and social capital shape an individual’s food and exercise practices in a community such as the Pakistani ethnic group in the UK.^16^

By incorporating Bourdieu’s practice theory into public health research, scholars and practitioners can gain a deeper understanding of how socioeconomic factors intersect with cultural and social influences in shaping food and exercise behaviours and health outcomes. This can inform the development of more comprehensive and contextually appropriate interventions and policies to address health disparities including CVDs among different socioeconomic groups such as Pakistanis in the UK. For example, a qualitative study done in Nottingham has introduced the idea of assumed shared food narratives to indicate how social networks operate to initiate or maintain certain healthy or unhealthy food practices.^21^

**Strengths and limitations:** To our knowledge, this is the first study using Bourdieu’s theory of practice to understand food as well as exercise practices in a community. Previous studies have mainly focussed on food practices. We collected rich data from two different qualitative methods. Our sample was diverse in the sense that it includes community leaders, first-generation and second-generation British Pakistanis, males and females of different age group, different levels of education and occupations. Importantly, we had participants from Mirpur and Punjab where most British Pakistanis originate.

Since we collected data in one UK city, and UK cities are heterogeneous, British Pakistanis living in other UK cities may have different access to food and exercise options, and hence different food and exercise practices.

## Appendix 1

### Characteristics of research participants

**Table 1.** Community leaders

S. No.	Age (years)	Education	Immigrant generation	Regional origin from Pakistan	Occupation
1	60	BA from Pakistan	First generation in the UK	Mirpur Azad Kashmir	Community development
2	40	BA from UK	Second generation in the UK	Punjab	Local and national Politics in the UK
3	68	Fifth grade from Pakistan	First	Mirpur	Community development
4	42	BA from Pakistan	Second	Mirpur	Community development
5	82	BA from Pakistan	First	Mirpur	Local Councillor

**Table 2.** Participants of focus group discussion (male)

S. No.	Age (years)	Education	Immigrant generation	Origin from Pakistan	Occupation
1	40	Bachelor degree from Pakistan	First	Punjab	Taxi driver
2	45	Tenth grade from Pakistan	First	Mirpur	Taxi driver
3	45	Tenth grade from Pakistan	First	Mirpur	Taxi driver
4	49	LLB from Pakistan	First	Mirpur	Taxi driver
5	36	Twelfth grade from Pakistan	First	Punjab	Taxi driver
6	38	Tenth grade from Pakistan	Second	Mirpur	Taxi driver
7	42	Tenth grade from Pakistan	Second	Mirpur	Businessman
8	40	Tenth grade from Pakistan	First	Mirpur	Painter
9	30	Twelfth grade from Pakistan	First	Mirpur	Takeaway
10	30	Twelfth grade from Pakistan	First	Mirpur	Car mechanic

**Table 3.** Individual interview participants (male)

Participant ID	Age (years)	Education	Immigrant generation	Occupation
1	41	GCSE from UK	Second	Takeaway
2	20	A level from UK	Second	Grocery shop
3	34	LLB from Pakistan	First	Taxi driver
4	20	A level from UK	Second	Student
5	34	PhD UK	Second	Pharmacist
6	45	Graduate from the UK	Second	Property advisor
7	32	BA from Pakistan	First	Takeaway
8	31	A level from UK	Second	Taxi driver
9	62	Tenth grade from Pakistan	First	Taxi driver
10	30	Tenth grade from Pakistan	First	Grocery shop
11	39	Tenth grade from Pakistan	Second	Restaurant
2	34	MSc from Pakistan	First	Grocery shop
13	47	BA from Pakistan	First	Taxi driver
14	52	Tenth grade from Pakistan	First	Taxi driver
15	29	MA from UK	Second	Teacher
16	71	Tenth grade from Pakistan	First	Pensioner
17	69	Tenth grade from Pakistan	First	Pensioner
18	64	MBA from Pakistan	First	Restaurant
19	42	LLB from Pakistan	First	Taxi driver
20	31	GCSE from UK	Second	Barber

**Table 4.** Focus group discussion participants (female)

Participant ID	Age (years)	Education	Generational status	Regional background	Occupation
1	40	GCSE from UK	Second	Mirpur	Support worker
2	79	None	First	Mirpur	Pensioner/housewife
3	33	GCSE from UK	Second	Mirpur	Housewife
4	45	None	First	Mirpur	Housewife
5	47	Fifth grade from Pakistan	First	Punjab	Housewife
6	74	None	First	Mirpur	Pensioner
7	58	Tenth grade from Pakistan	First	Punjab	Housewife
8	40	Fifth grade from Pakistan	First	Mirpur	Housewife
9	35	GCSE from UK	Second	Mirpur	Housewife
10	46	Fifth grade from Pakistan	Second	Mirpur	Housewife

**Table 5.** Individual interview participants (female)

Participant ID	Age (years)	Education	Immigrant generation	Occupation
1	39	GCSE from UK	Second	Beauty clinic
2	63	Midwife from Pakistan	First	Midwife
3	39	BSc from UK	Second	Psychologist
4	37	A level from UK	Second	Teacher assistant
5	29	Tenth grade from Pakistan	First	Housewife
6	39	A-Level from UK	Second	Office admin job
7	54	MBBS/medicine from Pakistan	First	Doctor
8	40	A level from UK	Second	Boots store
9	38	PGCE from UK	Second	Community worker
10	34	Degree from UK	Second	Accountant
11	51	GCSE from UK	Second	Support worker
12	52	GCSE from UK	Second	Health educator in primary care
13	43	GCSE from UK	Second	Office admin support job
14	57	GCSE from UK	Second	Community worker
15	57	GCSE from UK	First	Part-time shop work
16	45	O-level from UK	Second	Volunteer sector worker
17	30	O-level from UK	Second	Post Office worker
18	30	GCSE from UK	Second	Beauty clinic
19	35	A level from UK	Second	Supermarket worker
20	40	BA from UK	Second	Teacher
